# Stakeholder views on publication bias in health services research

**DOI:** 10.1177/1355819620902185

**Published:** 2020-02-03

**Authors:** Iestyn Williams, Abimbola A Ayorinde, Russell Mannion, Magdalena Skrybant, Fujian Song, Richard J Lilford, Yen-Fu Chen

**Affiliations:** 1 Reader in Health Policy and Management and Director of Research, Health Services Management Centre, University of Birmingham, UK; 2 Research Fellow, Warwick Centre for Applied Health Research and Delivery, University of Warwick, UK; 3 Professor of Health Systems, Health Services Management Centre, University of Birmingham, UK; 4 Patient and Public Involvement and Engagement Lead, Institute of Applied Health Research, University of Birmingham, UK; 5 Professor in Research Synthesis, Department of Population Health and Primary Care, University of East Anglia, UK; 6 Professor of Public Health, Warwick Centre for Applied Health Research and Delivery, University of Warwick, UK; 7 Associate Professor, Warwick Centre for Applied Health Research and Delivery, University of Warwick, UK

**Keywords:** health services research, publication bias, qualitative interviews

## Abstract

**Objectives:**

While the presence of publication bias in clinical research is well documented, little is known about its role in the reporting of health services research. This paper explores stakeholder perceptions and experiences with regard to the role of publication and related biases in quantitative research relating to the quality, accessibility and organization of health services.

**Methods:**

We present findings from semi-structured interviews with those responsible for the funding, publishing and/or conduct of quantitative health services research, primarily in the UK. Additional data collection includes interviews with health care decision makers as ‘end users’ of health services research, and a focus group with patient and service user representatives. The final sample comprised 24 interviews and eight focus group participants.

**Results:**

Many study participants felt unable to say with any degree of certainty whether publication bias represents a significant problem in quantitative health services research. Participants drew broad contrasts between externally funded and peer reviewed research on the one hand, and end user funded quality improvement projects on the other, with the latter perceived as more vulnerable to selective publication and author over-claiming. Multiple study objectives, and a general acceptance of ‘mess and noise’ in the data and its interpretation was seen to reduce the importance attached to replicable estimates of effect sizes in health services research. The relative absence of external scrutiny, either from manufacturers of interventions or health system decision makers, added to this general sense of ‘low stakes’ of health services research. As a result, while many participants advocated study pre-registration and using protocols to pre-identify outcomes, others saw this as an unwarranted imposition.

**Conclusions:**

This study finds that incentives towards publication and related bias are likely to be present, but not to the same degree as in clinical research. In health services research, these were seen as being offset by other forms of ‘novelty’ bias in the reporting and publishing of research findings.

## Introduction

Publication bias occurs when the direction or strength of research findings affects the likelihood of their publication,^1^ which can lead to distortions in syntheses of evidence on the effects of interventions or the association between variables. This, in turn, can prejudice decision making about, for example, the allocation of health care resources.^2^ While well documented in clinical research,^2,3^ little is known about publication bias in the reporting of health services research (HSR) and its impact on the evidence base and consequent decision making in this field. The lack of empirical investigation of publication bias is compounded by its absence from quality assessment procedures in many quantitative HSR evidence syntheses.^4,5^

Preliminary work on publication bias in quantitative HSR found the propensity for publication to be associated with the reporting of statistically significant or positive findings, and about one-quarter of HSR systematic reviews that used statistical methods for detecting potential publication bias finding evidence suggestive of its existence.^5,6^ These observations highlight the need for further empirical exploration and theorizing of the factors shaping publication outcomes. Explanations for the presence of publication bias make reference to publication ‘cultures’, in which competition for funding and pressure to publish in high impact journals combine to predispose academic researchers and journal editors to publish positive and/or statistically significant findings.^7^ However, there is lack of empirical investigation of the role of such ‘cultures’ in HSR. The role and influence of commercial conflicts of interest in how evidence is reported are also less well understood in relation to HSR.^8^ There is therefore a need for more in-depth investigation of publication and related bias in HSR.^9,10^ This is important not just for the scholarly maturation and objectivity of HSR but also for wider knowledge mobilization in health care improvement.^11^ Gaining a better understanding of the extent of publication bias and its determinants in HSR is a prerequisite for developing appropriate responses and adapting solutions applied in other research fields.^2^

This study explores stakeholder perceptions and experiences of the role of publication and related biases in quantitative HSR, which is defined as research relating to the quality, accessibility and organization of health services.^12^ Specifically, we sought to: understand the reasons for the (non-)occurrence of publication and related biases in quantitative HSR; identify the stages of the research-publication process that are most affected by publication bias and related biases, and why; and describe the strategies that are required to address publication and related biases in quantitative HSR.

## Methods

**Figure 1.**
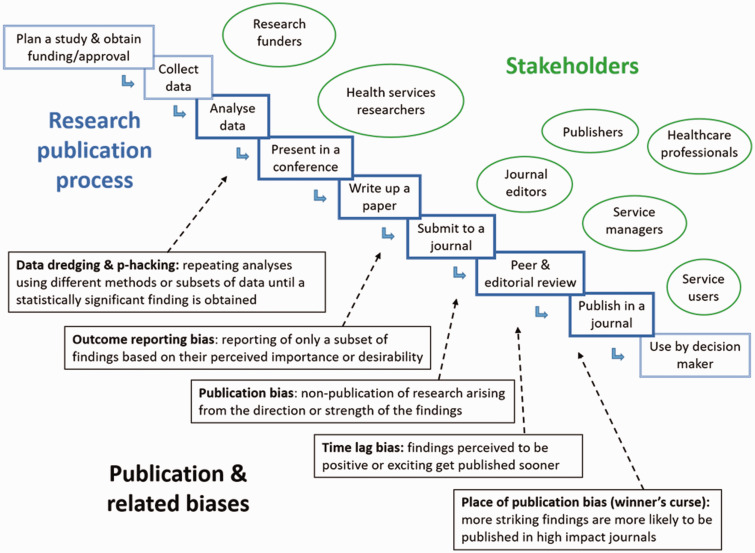
Forms of bias explored in the study.

Figure 1 illustrates the forms of bias explored in this study. They include bias that is likely to occur during the analysis and write-up stages of research, such as p-hacking, data dredging and selective outcome reporting, which is in the hands of those submitting manuscripts for publication. We were also interested in forms of bias that occur at the peer/editorial review and publishing stage, namely non-publication, slower publication or lower profile publication arising from the strength of direction of the findings.

### Study design and recruitment

We used semi-structured interviews with key informants responsible for the funding, publishing and/or conduct of HSR^13^ and health care decision makers as ‘end users’ of HSR, as well as a focus group with patient and service user representatives. Study participants were not required to have a specific research interest in publication bias, but were selected to include individuals (i) with a track record of quantitative HSR publication; (ii) who were at different stages of their careers (indicated by level of seniority) and/or (iii) who specialized in different HSR fields such as systematic review, improvement science, management, health sociology, health economics and operations research. The sample also included editors and assistant editors of medical and HSR-specific journals.

Our provisional sample target was between 20 and 30 participants to enable minimum coverage of all sample categories. The final sample comprised 32 participants, including 24 interviews and 8 focus group participants (Table 1).

**Table 1.** Study participants.

	Gender	First role	Second role	Country
Participant 1	M	Journal editor (medical)	Clinician	UK
Participant 2	F	Journal editor (medical)	n/a	UK
Participant 3	M	Junior-mid researcher	n/a	Germany
Participant 4	F	Journal editor (HSR)	Senior researcher	UK
Participant 5	M	Mid-senior researcher	Journal editor (HSR)	UK
Participant 6	F	Senior researcher	Journal editor (HSR)	UK
Participant 7	M	Senior researcher	n/a	UK
Participant 8	M	Mid-senior researcher	n/a	UK
Participant 9	F	Junior-mid career researcher	n/a	UK
Participant 10	F	Senior researcher	n/a	UK
Participant 11	M	Journal editor (HSR)	Senior researcher	UK
Participant 12	M	Senior researcher	Clinician	UK
Participant 13	F	Journal editor (Medical)	Clinician	USA
Participant 14	M	Senior researcher	Research funder	UK
Participant 15	M	Consultant evaluator	Senior researcher	UK
Participant 16	M	Research funder	Senior researcher	UK
Participant 17	M	Research funder	Junior-mid researcher	UK
Participant 18	F	Research funder	Senior researcher	UK
Participant 19	F	Senior researcher	Journal editor (HSR)	UK
Participant 20	F	Manager	n/a	UK
Participant 21	F	Journal editor (HSR)	Senior researcher	Canada
Participant 22	M	Senior researcher	Clinician	Canada
Participant 23	M	Journal editor (medical/HSR)	Senior researcher	USA
Participant 24	M	Manager	Clinician	UK
Focus group participant 1	M	Patient/service user representative	n/a	UK
Focus group participant 2	F	Patient/service user representative	n/a	UK
Focus group participant 3	M	Patient/service user representative	n/a	UK
Focus group participant 4	F	Patient/service user representative	n/a	UK
Focus group participant 5	F	Patient/service user representative	n/a	UK
Focus group participant 6	M	Patient/service user representative	n/a	UK
Focus group participant 7	M	Patient/service user representative	n/a	UK
Focus group participant 8	F	Patient/service user representative	n/a	UK

The research sample was drawn predominantly from the United Kingdom (UK), with a small number of international respondents also included where these were considered to strengthen and/or enrich the dataset. Three respondents were included primarily for their roles at major funders of UK HSR research, and two participants were included as national and local decision makers within the English National Health Service. Although sampled according to the above characteristics, many participants were also able to draw on other experience relevant to our study (‘primary’ and ‘secondary’ roles in Table 1). As noted, we conducted a single focus group with patient and service user representatives, recruited from existing UK organizations and networks. The focus group format was selected to enable participants to clarify and develop their thoughts and views; patient and public experts in the project team (MS, IW) believed that a focus group approach would aid insight and support discussion in relation to what is a somewhat specialist topic.^14,15^

The research team, in consultation with the Study Steering Committee and Project Management Group, drew up lists of potential interview participants based on the sample characteristics described above and worked through these until all categories were covered. Potential participants were invited via email using publicly available email addresses, with a follow-up email sent to non-responders. Of the 27 invited respondents that did not take part, eight cited lack of available time, four indicated a lack of interest/expertise in the topic and 15 did not respond. Those declining to take part were similar in profile (e.g. gender, role, location, seniority) to the included sample and no obvious bias can be inferred from their absence from the sample.

### Data collection and analysis

We obtained ethical approval from the University of Warwick Biomedical and Scientific Research Ethics Committee (REGO-2017-1918 AM01). Topic guides for the interviews and focus group were informed by previous phases of the study and focussed on key informants’ perceptions and past experience of publication bias in HSR, and their views on possible approaches to its mitigation. Interview and focus group data were collected during September 2017 to August 2018; interviews were conducted by the lead author and the focus group facilitated by the lead author and one co-author, both senior researchers experienced in conducting qualitative research. As researchers in related fields, some of the interview participants were known to the research team although we detected no obvious resulting interviewer effects in interviews. All participants opted for a telephone interview format and interviews were undertaken one-on-one; they ranged from 20–45 min in length and the focus group lasted 1.5 h.

Data were analysed inductively using the framework provided by the research aims and existing literature, as well as issues identified in prior phases of the research. Interviews were fully transcribed, and we used qualitative coding software (NVIVO Version 11) to facilitate data storage and retrieval during analysis. Two members of the research team contributed to initial building of thematic coding frames from the data and independent coding of a data subset. Four team members then applied a revised coding frame to a subset of transcripts, and reviewed these further following comparison. All transcripts were then coded by the lead researcher and checked by a second. Identified themes were discussed at meetings of the core project team and the study steering group, which included patient and public involvement experts, health system decision makers and researchers. Saturation checks conducted during the final three interviews suggested that while additional themes of interest were still forthcoming, these did not relate to the core research aims.^16^ In this paper, these are put forward as areas for possible future investigation. This follows a summary account of the findings, accompanied by illustrative quotations, and a discussion of themes.^17^

## Results

The first issue we explored with interview participants was the extent to which they believed that publication and related bias are a feature of HSR. Many felt unable to respond with certainty. Of the 16 participants expressing a view, 11 believed such biases to be prevalent, whereas 5 believed them to be rare. However, within each of these groups, there were varying levels of confidence in this assessment. For example, in the former group, while some argued bias to be ‘rampant’, others were more cautious. Those in the latter group were also hesitant in their judgement. Subsequent findings therefore should be qualified by this underlying uncertainty.

### Funding of quantitative HSR

The circumstances in which HSR is funded was widely agreed to have a bearing on the likelihood of publication and related bias. Many participants believed the risk of bias to be lower in highly formalized programmes of research funding that involve criteria-based decision making by independent commissioning panels and that use external peer review (for example, the UK National Institute for Health Research (NIHR), Health Services and Delivery Research Programme). It was further noted that these funders frequently mandate full reporting and require data to be made publicly available. Other funder characteristics that were perceived to lower the risk of publication and related bias included the presence of conflicts of interest policies and requirements for adherence to study protocols. Larger research projects led by teams with a strong academic track record (and motivated to produce academic outputs) were also seen as being at lower ‘risk’ of publication and related bias. Funders in these circumstances were described as being largely ‘blind’ to considerations other than the rigour of research outputs.

With the NIHR or research councils, you know, you put in your research proposal and they let you get on with the research. (Participant 12, senior researcher/clinician UK)

This model was contrasted with end user sponsored research, where funders have a direct interest in the results and engage in ongoing interaction with the research team. One participant for example noted that government bodies directly commissioning evaluations of their own programmes were often *‘desperate for those to be shown to be positive’*, and one other participant spoke of charitable foundations requiring *‘striking’* findings in order to *‘feed their communications machine’*. However, most participants emphasized the relative autonomy of those conducting research in such circumstances and indicated that any funder ‘pressure’ was likely to be ‘indirect’.

What I worry about is this subtle, indirect pulling of punches; so softening the edges of the messaging. Because you get comments coming back from policy officials saying things like ‘you’ve given us a kind of glass half empty story. Can’t you turn it round?’ And of course, often it is a matter of language and emphasis. (Participant 11, editor/senior researcher, UK)

All participants that expressed a view felt that pressure from funders was of a lesser magnitude than that generated in clinical research, based on their experience:

There’s a danger of framing it in the same way as in medical research because the stakes are very high in medical research and there’s big commercial involvement through the pharmaceutical companies, who’ve been repeatedly caught ‘off side’, as it were. Whereas I think the same issues don’t apply, at least universally, in health service research. (Participant 16, funder/senior researcher, UK)

However, the lack of formal requirement for peer reviewed publications from such government bodies was identified to increase the risk of partial or non-publication of study findings.

It effectively leaves the onus on getting peer reviewed publications up to us which is exactly when publication bias is more likely. (Participant 14, senior researcher/funder, UK)

It was noted that local evaluations and audits are seldom designed to be reported beyond the immediate organizational context, and that therefore it was inappropriate for them to be subjected to the rigours of formal peer review before publication. In such cases, publication bias was seen to result from the inappropriate publishing of data outside of the intended setting. Participants reflected on the dividing lines between research and other forms of knowledge:

I think the boundary between research and service improvement is really blurred and if you’re just using someone’s routinely collect[ed] data, not purposely collected for a research study, but just kind of scraped off their systems, and you’re using it for a model which will help them improve their systems, then I don’t call that research personally. (Participant 4, editor/senior researcher, UK)

Another participant identified the source of ‘editorial control’ as the primary differentiation between research data and consultancy in a previous role:

We did both consultancy and research, and the distinction was research meant that there was an explicit agreement up front, that we the researchers would be able to have editorial control so that we could seek publication in a journal. In other words, that the funder could not stop us publishing. (Participant 15, consultant/senior researcher, UK)

### Publication bias in journal decision making

Participants reflected on the extent of publication bias in journal editorial decisions on whether to accept or reject HSR submissions, with researchers most likely to argue that publication bias was pervasive in academic publishing:

I think *[positive effects]* are of interest to all the journals and some are more explicit about it than others … All of the journals, despite their veneer of academic impartiality, are highly news oriented and they are kind of pushing for space in that crowded market place. (Participant 16, funder/senior researcher, UK)

You would never ever send something non-significant to a top journal, that’s my feeling. (Participant 3, junior-mid career researcher, Germany)

Study participants described a range of other characteristics they thought would predispose journal editors to accept HSR manuscripts for publication, including study scale; extent of methodological and theoretical innovation; and political salience of the topic/intervention. Along with methodological rigour, these factors were seen to reduce the influence of the magnitude and statistical significance of quantitative findings, especially in HSR journals (while attributed higher importance in medical journal decision making). These perceptions would suggest that HSR journals are more susceptible to other forms of ‘novelty’ bias than they are to publication bias driven by the magnitude and statistical significance of findings.

There was some disagreement about the role played by editors and peer review in publication bias. While some editors placed faith in peer reviewers’ ability to detect publication bias (‘*they are our human funnel plots*’), others felt that awareness of publication bias could be increased:

Certainly, as a reviewer and as an editor I don’t see many questions or prompts asking about the presence of publication bias. I don’t see many reviewers’ comments about publication bias unless they’re aware of particular studies that have been missed. (Participant 5, mid-senior researcher/editor, UK)

In general, the proliferation of peer reviewed journals was seen as reducing the risk of publication bias, for example in HSR systematic reviews.

### Researcher conduct in quantitative HSR

Participants were asked for their views and experiences concerning researcher behaviour in the process of analysing data and writing for publication. Responses were mixed within and across categories of stakeholder groups. Some argued that in trial-based research, behaviours such as selective reporting and ‘data hacking’ were no longer possible, while others believed these behaviours to be commonplace. Interviews suggested that the extent of publication bias was linked to research design with, for example, service evaluations at lower risk than other forms of association studies.

The more subtle and more common type *[of publication bias]* is the issue around hypothesis testing when you’re the statistician doing the analysis and when you do a lot of what you would, in your mind, neatly label ‘preliminary analysis’; a lot of unofficial hypothesis testing – ‘see if this works’ – and subgroup analysis … That is a massive problem. (Participant 8, mid-senior researcher, UK)

It was clear from the interviews with researchers that, to the extent to which they considered such biases to be present, this was driven by the perceived expectations of journals and their own academic institutions. Some expressed a concern that ‘job prospects’ in academia depended on high impact journal publication, and it was notable that each of the more junior researchers believed publication bias to be widespread, whereas some senior researchers reported little pressure to publish positive results.

Almost all of my studies show that whatever I’m looking at doesn’t work … The answer to your question is that, in my own personal research, it would not cross my mind not to publish something. (Participant 12, senior researcher/clinician, UK)

However, there was general support for the view that research teams might take longer to submit negative or null results for publication:

I’ve been involved in a couple of large implementation research trials and neither of them came out with positive or significant outcome findings … To motivate yourself to get that negative finding trial out there, it’s a real challenge. I think that there is something about not wanting to share something that you feel failed in some way, which is ridiculous. (Participant 18, funder/senior researcher, UK)

Some respondents identified a risk of publication bias where evaluators were responsible for developing the intervention and many felt publication bias was a risk within much service improvement research:

There’s strong movement within quality improvement, which is a very enthusiastic kind of movement, one that celebrates success in lots of ways. And some relatively large claims are made for some work that’s often done with relatively simple ways of looking at data … Now that is all maturing rapidly but my instincts are that because of that strong enthusiasm and the desire for things to be seen to work, there’s a risk. (Participant 17, funder/junior-mid researcher, UK)

This problem was seen by the two managers in our sample to be compounded by a health care environment that rewarded ‘good news’ stories about health care innovations and improvements. Table 2 summarises the factors seen to increase or decrease the risk of publication bias in HSR.

**Table 2.** Predisposing factors for research publication and its related biases.

Stage	Reduced risk	Increased risk	Explanation
Commissioning of HSR	– Criteria-based decisions – Autonomous panels – External peer review – Publication requirements – Conflicts policies – Study protocols	– Non-criteria-based allocation of funding – Iterative research design – Ongoing interaction with research teams – Funder involvement/interest in intervention – No publication requirements	Factors such as criteria-based funding decisions and autonomous peer review and decision making were seen as reducing the avenues through which sponsors of research could influence study aims and researcher conduct. These factors were linked to the use of study protocols and an ‘arms-length’ relationship between funding bodies and research teams. The absence of these factors was associated by some interview participants with an increased risk of funder influence over content and rates of publication. Funders may have a vested interest in the success of an intervention being evaluated, either professionally or financially; for example, governmental and other bodies that commission evaluations of their own interventions may have a direct interest in the presentation and reporting of results
Conduct of HSR	– Prospective trial-based design – High incentives to publish – High research experience/expertise – Absence of author involvement/interest in intervention studied	– Retrospective association studies – Low incentives to publish – Low research expertise – Presence of author involvement/interest in intervention studied	Non-evaluation association studies were seen as more susceptible to data dredging than evaluation studies which were more likely to follow a trial-based design. The latter were perceived as more likely to involve prospective specification of research aims and methods, as well as being more likely to be subject to stringent review against study protocols Researcher expertise, in combination with other factors, was seen as an important check on author over-claiming and university-based researchers in general were seen as more likely to wish to publish findings irrespective of their strength or direction. As with funders, risks were identified where researchers had a vested interest in the intervention being evaluated
Publishing HSR	– HSR submissions to HSR journals – Low institutional pressure levels – Publications contain other ‘novel’ features	– HSR submissions to medical journals – High institutional pressure levels – Publications contain no other ‘novel’ features	Some participants distinguished between medical journals and HSR journals when assessing the likelihood of publication bias. The logic was that effect sizes/statistical significance are of greater interest to medical journals, especially where the intervention does not have a clinical outcome, as is the case for most HSR. This meant that institutional pressures to publish in high impact (i.e. medical) journals were considered an important factor. By contrast, HSR journals were seen as valuing other forms of ‘novelty’ alongside strength and direction of findings

### Distinctive features of HSR

In describing their own work, a handful of the more senior researchers emphasized the characteristics of some HSR that, they argued, made it less susceptible compared to clinical research to publication bias. These included: the complex nature of some HSR interventions and the uncertainty this creates in measurements of effects; the role of context in determining associations and intervention outcomes, which reduced the possibility of randomization and study replication; the tendency of HSR studies to address multiple research questions, so that considerations of effects and associations become merely one of many sources of reported data

I wonder whether epistemologically they are doing different things; essentially within the *[Health Technology Assessment]* community it’s much more of an aggregative process coming up with, you know, pooled overall effects, etc. But if you assume that there will be a great deal of heterogeneity in health service delivery research then perhaps epistemologically you’re trying to do much more about making sense of options and mapping different models. And linked to that is the interplay of context so that within an HTA context there’s almost the assumption that a drug will work in a relatively similar way across multiple contexts, whereas a service delivery intervention is very context-specific. So maybe people are bringing different quality markers or a different sort of epistemological view. (Participant 5, mid-career researcher/editor, UK)

Some participants cited the uncertainties that characterize many HSR studies (for example, relating to interventions, contexts and outcomes) as one reason for such research having a somewhat lower profile than clinical research, and this may be subject to less scrutiny from, for example, media, government and industry. These views were reflected by attitudes towards pre-registration of studies and the strict application of protocols, which have been advocated elsewhere as a means of preventing publication bias.^18^ Thus, while many supported their adoption into HSR, others believed this would result in unwarranted and inappropriate constraints on researcher conduct. As well as the practical challenges these would impose on large, multi-strand studies, participants argued that they would compromise sensitivity to changes in context during the lifetime of a research programme.

## Discussion

Overall, a notable finding of the study is that many study participants felt unable to say with any degree of certainty whether publication bias represents a significant problem in HSR. Although the majority believed it to be present, much of this was based on what they would expect rather than what they had personally observed. Our findings point to factors that may increase or reduce the risk of publication and related bias, rather than actual occurrences. Nevertheless, the study appears to mirror the empirical findings of our previous work,^4,5^ while offering useful further qualitative insights.

HSR as an academic field contains multiple sub-specialisms which do not cohere into a unified whole. This is reflected in the diversity of theoretical and empirical traditions, and in the range of options for researchers seeking to publish their work. Many of our study participants reported institutional pressures to publish in journals with high impact factors, which led them to target medical journals. However, this was widely agreed to be challenging for HSR. The preference, on the part of researchers and journal editors, for significant (if not necessarily positive) results was seen to be exacerbated as a result. Although journal hierarchies were felt to exist in HSR, these were seen as more diffuse, spanning a range of sub-disciplines. Furthermore, HSR journals were believed to apply different criteria to medical journals when accepting or declining manuscripts for submission. This may also reflect the lower average impact factor for HSR journals compared to medical journals.

The disciplinary heterogeneity of HSR may account for the variety of views expressed concerning the pervasiveness of publication and related biases. It also has implications for boundary delineation between HSR and other forms of knowledge, which participants suggested were often unclear. For example, they drew broad contrasts between externally funded and peer reviewed research on the one hand, and end user funded quality improvement projects on the other, with the latter perceived as more vulnerable to selective publication and author over-claiming.

A key area of disagreement among study participants was in the attitudes to the adoption of guidelines to address publication bias. Many were supportive of pre-registration and using protocols to pre-specify study outcomes, but others saw this as an unwarranted imposition on their autonomy as researchers. Although this might reflect differences between disciplines contributing to HSR, there was general agreement that HSR studies often contained more than summative assessments or measurements of associations. The prevalence of multiple study objectives, indefinite interventions, moderating contextual factors and a more general acceptance of ‘mess and noise’ in the data and its interpretation, apparently reduced the importance attached to replicable estimates of effect sizes. Some interview participants perceived the relative absence of external scrutiny from manufacturers of interventions or health system decision makers to add to this general sense of ‘low stakes’ in the field of HSR. However, this acceptance of variation will make the task of evidence synthesis and meta-analysis more difficult.

Overall, interview participants considered incentives towards publication bias to be present but not to the same degree as in clinical research. Our study suggests there are pockets that might be particularly vulnerable to publication bias within HSR, for example in retrospective decisions to submit for publication of data that have been gathered primarily for other purposes. It also suggests that forms of bias may be linked to study type; for example, non-evaluation association studies may be more susceptible to p-hacking and selective outcome reporting, whereas evaluation studies may be subject to greater degrees of funder pressure. More broadly, however, the implications of our study are that the direction and strength of results compete with other potential sources of ‘novelty’ in influencing and shaping publication intentions and outcomes.

The study sheds some light on considerations of ‘impact’ as a determinant of publication patterns in HSR and other research. This was seen as potentially compromising researcher and journal behaviour, but mainstream research funding bodies are also under pressure to maximize the impact of the research they commission, although this was not raised in our sample. The ‘research cultures’ evoked in our interviews share some features of those described previously, including most notably the pressure to publish in high impact journals.^7^ In comparison to clinical research, HSR tends to be more fragmented, and the presence of ‘sub-cultural’ variation may attenuate some of the drivers of publication bias. However, again some of these sub-cultures, notably the growing field of service improvement research, appear to be more susceptible than others.

### Limitations and future research

This study drew on an expert sample and as such is not intended to be generalisable to the wider community of HSR researchers, funders and stakeholders. Triangulation and/or large-scale investigation is therefore required before definitive claims can be made as to the presence and impact of publication and related biases in HSR. It would be fruitful to explore further the various research HSR sub-cultures. Other variables might also be explored; for example, senior researchers appeared more confident than their junior equivalents of successfully submitting negative or neutral findings for peer review publication. Furthermore, the interviews suggested the need for examination of other sources of ‘novelty’ in the publication of HSR, such as study scale, extent of methodological and theoretical innovation, and political salience of the topic/intervention. Given the multiple disciplines contained within HSR and their frequent departure from a biomedical research paradigm, it is not clear that the language of ‘bias’ is suitable to frame such investigation, especially where this includes qualitative research.^19^

Our primary interest in this paper has been the phenomena of publication and related biases in the production and reporting of HSR. Not surprisingly, the interviews conducted with service managers, and the focus group with patients and service users, showed them to be more concerned with the ultimate impact of bias on services and patients. We were unable to give space here to explore the impact of bias on subsequent stages of the evidence-into-practice process, but consider addressing the potential for publication bias in HSR to be a critical factor in the next generation of evidence-based health care.^11^
